# Occupational exposure to inhalational anesthetics and the risk of spontaneous abortion: a systematic review and meta-analysis

**DOI:** 10.3389/fpubh.2026.1766912

**Published:** 2026-04-01

**Authors:** Tang Ying, Wang Yuanqing

**Affiliations:** 1 Department of Anesthesiology, The Third People’s Hospital of Deqing, Huzhou, China; 2 Health Management of Huzhou Health Service Center, Huzhou, China

**Keywords:** abortion, anaesthetic gas, meta-analysis, occupational health, systematic review

## Abstract

**Background:**

Healthcare professionals, particularly anesthesiologists, nurses, and dental staff, are routinely exposed to inhalational anesthetic agents such as nitrous oxide and halogenated gases. Although scavenging and ventilation systems have reduced ambient levels, concerns remain regarding reproductive risks associated with chronic exposure. Previous studies have yielded inconsistent results. A systematic review and meta-analysis of published observational studies was conducted to clarify the relationship, assessing reproductive outcomes among healthcare workers who were exposed.

**Objective:**

This systematic review and meta-analysis aimed to clarify the association between occupational exposure to anesthetic gases and the risk of spontaneous abortion among healthcare workers.

**Methods:**

A systematic search was performed in PubMed, Scopus, and Web of Science up from inception until the end of July 2025. Observational studies assessing spontaneous abortion among female healthcare workers exposed to inhalational anesthetics were included. Two reviewers independently screened and extracted data and assessed study quality using the Newcastle–Ottawa Scale (NOS). Pooled odds ratios (ORs) and 95% confidence intervals (CIs) were calculated using a random-effects model. Heterogeneity was evaluated using the I^2^ statistic, and potential moderators were explored via subgroup and meta-regression analyses.

**Results:**

Seventeen studies were included. The pooled analysis showed 1.29-fold higher odds of spontaneous abortion among exposed workers compared with unexposed controls (OR = 1.29; 95% CI: 0.96–1.75; I^2^ = 87%). Subgroup analyses indicated stronger associations in North American studies and among high-exposure occupations. Meta-regression analysis indicated that exposure duration is a significant predictor (*p* = 0.010), accounting for 73.6% of the heterogeneity. Sensitivity and Egger’s tests revealed robust findings without evidence of publication bias.

**Conclusion:**

Occupational exposure to anesthetic gases may increase the risk of spontaneous abortion, particularly in those who are at high exposure, emphasizing the need for stringent exposure control and improved workplace safety standards.

## 1 Introduction

Inhalational anesthetic agents, including nitrous oxide, halothane, isoflurane, sevoflurane, and desflurane, are the main drugs used in both induction and maintenance of general anesthesia as part of the surgical procedure. Even though inhalational anesthetic agents have become an established part of our practice, there are a variety of concerns that have emerged regarding possible reproductive side effects resulting from prolonged occupational exposure, specifically, the elevated risk for of spontaneous abortion and congenital malformations are of essential concern to women of reproductive age working in operating areas (1–3).

Before 1990, other factors could have added to a greater ambient level of anesthetic gas in the operating room. For example, periodically before 1990, scavenging systems and adequate ventilation systems all effected a greater decrease in ambient anesthetic gas than what previously existed (4). While some recent reports have apprehensively concluded that there is no difference between current occupational exposure to anesthetic gas and spontaneous abortion, there evidences of a significant positive relation between exposure to such gases and abortion (2, 5, 6). Additionally, previous epidemiologic studies showing a potential ‘increased risk’ of spontaneous abortion in people exposed to inhaled anesthetics typically involved poor follow-up and were undertaken before the common adoption of scavenging systems and good ventilation, which could relate to increased ambient anesthetic gas levels (5). In addition, some significant studies, including the meta-report published by Boivin in 1997, are older and may not accurately reflect exposures or practices of the present-day workplace or dental operating room setting (1). Although previous reviews imply an association between inhalational anesthetics and spontaneous abortion, heterogeneity and limitations of the included studies prevent a firm conclusion (7). These reviews suggest that the absence of exposure controls or prolonged exposure times, especially in lower-resource settings, may introduce significant exposures (7, 8). Nevertheless, these reviews explicitly declared that have reached no definitive conclusion on risk of spontaneous abortion in health workers exposed to inhalational anesthetics. Subsequently more reviews, particularly meta-analysis that poses a statistical method and can reach definitive results are warranted to clarify this matter.

The latest European directive focusing on reprotoxic agents is directive (EU) 2022/431, which extends strict carcinogen-level occupational protections to reprotoxic substances, especially nitrous oxide due to its classification as “Toxic to Reproduction” (Category 1B). These regulatory forces healthcare facilities to manage nitrous oxide under CMR (Carcinogens, Mutagens, Reprotoxic) framework, mandating the application of the “STOP” hierarchy—prioritizing substitution where feasible, enforcing strict technical measures (like scavenging systems) to minimize exposure levels, and keeping detailed records of exposed staff. Additionally, Directive (EU) 2022/431 explicitly bans the use of Desflurane as of January 1, 2026, except for strictly necessary medical cases, due to its high Global Warming Potential. Conversely, other volatile agents like sevoflurane generally remain outside this specific directive’s rule unless strictly classified as reprotoxic. In general, recent European directives, exposure to anesthetic gases is forbidden for pregnant women throughout the entire pregnancy and after delivery until the end of breastfeeding, due to the well-recognized chemical risk associated with occupational exposure among healthcare workers (HCWs). (9)To ensure occupational safety around inhaled anesthetics, the dose–response relationship is crucial for assessing the risk of adverse events in the workplace. Several countries have recommended different exposure limits (10); the US National Institute for Occupational Safety and Health (NIOSH) guidelines provide a time-weighted average of 25 ppm of nitrous oxide and 2 ppm of halogenated anesthetics (those containing isoflurane, halothane, enflurane, and sevoflurane) (11).

We conducted this systematic review to resolve the discrepancies among previous original and review studies. We aim to clearly communicate the association between exposure to inhalation anesthetics and miscarriage risk, and to offer reasons as to why workplace health policies should protect the reproductive health of healthcare workers (12). This apparent contradiction established a relevant gap in the literature, indicating that a systematic review and meta-analysis that merged the most up-to-date scientific evidence, as we provided, was necessary. We aim to further clarify the association between occupational exposure to inhalational anesthetics and the risk of spontaneous abortion, and to provide a rationale for occupational health policies that protect the reproductive health of health care workers.

## 2 Methods

We adhered to the PRISMA guidelines in all steps of preforming this systematic review (13).

### 2.1 Search strategy

A comprehensive literature search was conducted in PubMed, Web of Science, and Scopus electronic databases, along with manual searches for studies in additional sources published from inception up to the end of July 2025. The following search strategy was applied for searching in the PubMed: (“anesthetic gases”[tiab] OR “anaesthetic gases”[tiab] OR “inhalational anesthetics”[tiab] OR “inhalational anaesthetics”[tiab] OR “waste anesthetic gases”[tiab] OR “Nitrous Oxide”[Mesh] OR “Anesthetics, Inhalation”[Mesh] OR nitrous oxide[tiab] OR halothane[tiab] OR isoflurane[tiab] OR sevoflurane[tiab] OR desflurane[tiab] OR enflurane[tiab]) AND (“Abortion, Spontaneous”[Mesh] OR “spontaneous abortion”[tiab] OR miscarriage[tiab] OR “reproductive outcomes”[tiab] OR “congenital abnormalities”[tiab] OR teratogenesis[tiab]. A complete list of all search terms used in databases is located in the Supplementary file.

### 2.2 Inclusion and exclusion criteria

The inclusion criteria for studies are as follows: the study population must consist of women of reproductive age who have had documented occupational exposure to inhalation anesthetics, including nurses, anesthesiologists, dentists, and veterinarians. The comparator group must consist of women who are unexposed or have a lower occupational risk related to inhalation anesthetics. The primary outcome of interest is spontaneous abortion or miscarriage. Study designs that were included as eligible are observational studies (cohort, case–control, and cross-sectional), as well as randomized controlled trials when applicable. Publications were limited to those published in English or Chinese.

Exclusion criteria included review articles, case studies, conference abstracts, and protocols etc. Additionally, studies on animals, studies with an inappropriate control or comparison group, and studies not published in English or Chinese were excluded. Exposure of interest is occupational, and studies assessing clinical (but not occupational) exposure to inhalation anesthetics were excluded. The PECO framework in this study is as follows:

- P (Population): Female healthcare workers (e.g., anesthesiologists, nurses, etc.).
- E (Exposure): Occupational exposure to inhalational anesthetic agents (e.g., nitrous oxide, halogenated gases).
- C (Comparator): Female in general population or healthcare workers with no occupational exposure to inhalational anesthetics.
- O (Outcome): Occurrence of spontaneous abortion.

### 2.3 Data extraction

Two independent reviewers assessed titles and abstracts for eligibility, and the full text was reviewed for potentially relevant studies. Any disagreements by reviewers were resolved through discussion, and if necessary, by a third reviewer. For each included study, we extracted the following data: study design, population characteristics, exposure measures and methods of assessment, a definition for and ascertainment of spontaneous abortion, risk estimates (and 95% confidence intervals), and any adjustments for confounders.

### 2.4 Statistical analysis

The meta-analysis was performed in RevMan, version 5.3, and STATA version 18. We combined effect estimates (for example, odds ratios or relative risks) along with the standard errors of these estimates in a random-effects model. The heterogeneity across studies was determined using the I^2^ statistic. We reported the pooled estimates and 95% confidence intervals. We conducted sensitivity analyses and individually omitted each study and evaluated the stability of the findings to study omission. Publication bias was assessed visually by funnel plots and statistically with Egger’s regression test. We adopted the conventional significance level of *p* < 0.05 and declared statistically significant based on this threshold.

### 2.5 Quality assessment

The risk of bias for the included studies was independently assessed by two authors using the Newcastle–Ottawa Scale (NOS) method. NOS indicates risk of bias across three domains: selection, comparability, and outcome (or exposure), and stars can be given based on the quality of the studies up to a maximum of 9 stars. NOS was designed for observational studies (e.g., cohorts, cross-sectional studies). Any differences between the two authors were resolved by discussion among the authors and re-examination. Risk of bias assessment visualized using Critiplot webtool (14).

## 3 Results

### 3.1 Literature search and study characteristics

**Figure 1:**
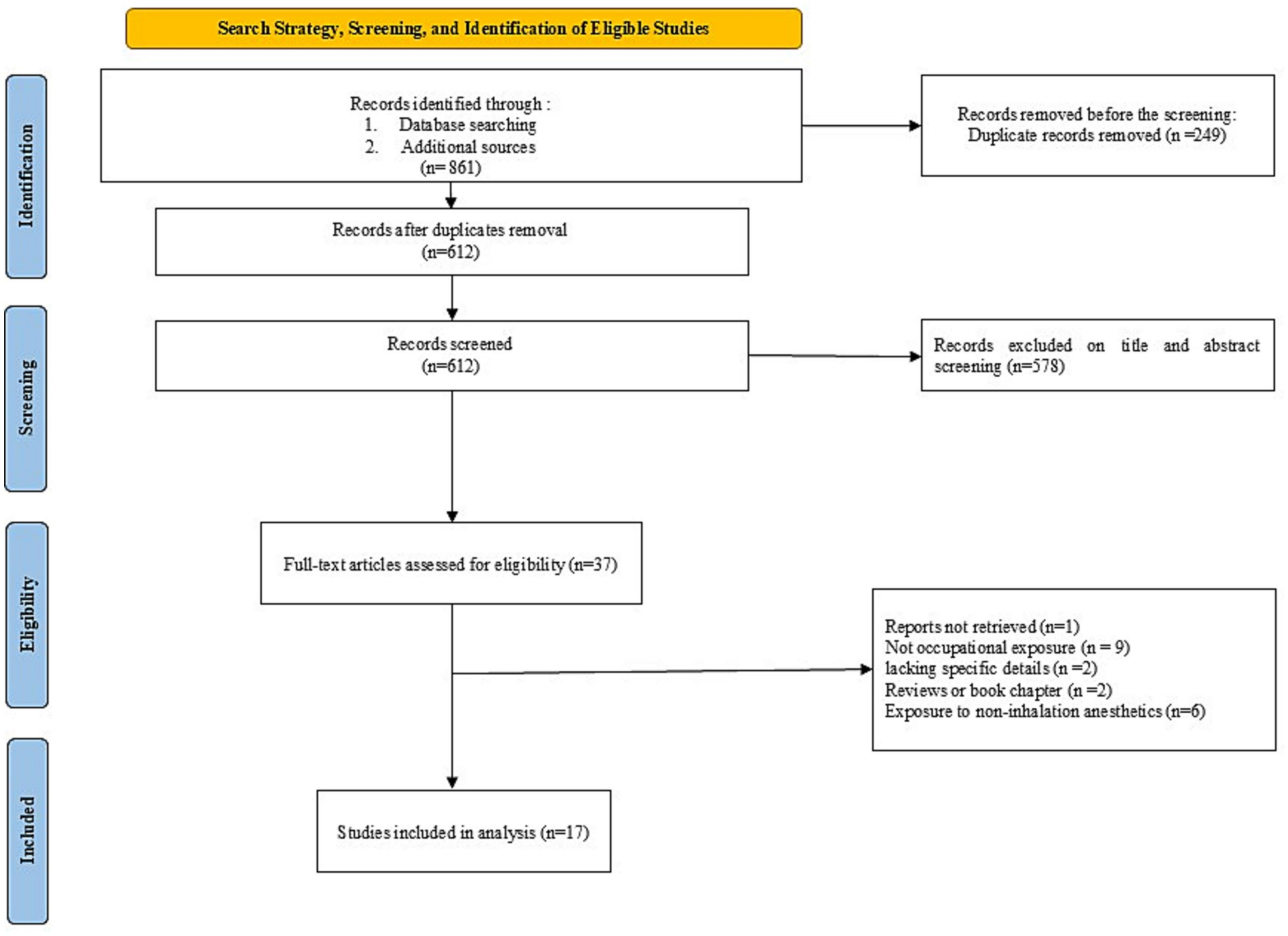
PRISMA flow diagram illustrating the study selection process, from initial identification of records to the final inclusion of studies in the meta-analysis.

Initial searches yielded 861 records identified through database searching and additional sources. After removing 249 duplicate records, 612 unique records remained for screening. Following title and abstract screening, 578 records were excluded, and the remaining studies underwent full-text review. Ultimately, 17 studies (2, 3, 15–29) met the inclusion criteria and were included in the final meta-analysis. A summary of study characteristics is presented in Table 1, and the study selection process is illustrated in the PRISMA flow diagram (Figure 1).

**Table 1:** Characteristics of studies included in the systematic review and meta-analysis on workplace exposure to inhalation anesthetics and spontaneous abortion.

Study	Type of study	Location	Studied population	Exposure levels	Monitoring methodology	Anesthetic exposure	Main result	Confounders
Nazanin Izadi et al. (2024)	Cross-sectional	Iran	Total of 733 female healthcare workers (married ≥1 year, ≥3 years work experience). Control Group: Internal comparison within the cohort	Self-reported chemical exposures	Self-reported questionnaire	Anesthetic gases (general)	Chemical exposures (solvents) and ergonomic factors were identified as risk factors for adverse outcomes (stillbirth, spontaneous abortion).	Smoking and alcohol consumption
Megersa Kelbesa Olika et al. (2022)	Comparative Cross-sectional	Ethiopia	Total of 292 female healthcare workers in operating rooms. Exposed: 146 Unexposed: 146	Defined by workplace location	Questionnaire	Mixed inhalational anesthetics (N₂O, halothane, isoflurane, sevoflurane)	Comparison of exposed operating room staff vs. unexposed staff often shows a higher prevalence of adverse reproductive outcomes (spontaneous abortion, menstrual disorders) in the exposed group, typically attributed to lack of scavenging systems in the study setting (Ethiopia).	History of alcohol, smoking, and contraceptive use (in some cases)
Bilijana Eftimova et al. (2017)	Cross-sectional (Case–Control)	Republic of Macedonia	Total of 43 participants: Case Group: 20 Staff from the Department of Anesthesiology and Intensive Care Control Group: 23 Staff from the Department of Internal Medicine	Time-Weighted Average (TWA) measured over 8-h shifts	Environmental Monitoring: Continuous measurement of N₂O concentration in the breathing zone using a handheld electrochemical instrument with data logging.	Nitrous Oxide (N₂O)	The exposed group had significantly higher rates of headaches, dizziness, nausea, vomiting, euphoria, and tachycardia compared to controls. Chronic exposure was associated with adverse health symptoms.	NA
Amrutha Nagellla et al. (2015)	Cross-sectional	India	Total of 1,563 complete responses: Case Group: 345 female anesthesiologists Control Group: 611 spouses of male anesthesiologists (serving as the unexposed/less exposed comparison group)	Self-reported usage frequency	Online questionnaire/survey	Nitrous oxide (used by 90%), halothane, isoflurane, sevoflurane	Female anesthesiologists working in the OR during the first trimester had a significantly higher incidence of spontaneous abortion and birth defects compared to those who did not work in the OR or the general population.	NA
Sandra I. Allweiler (2013)	Cross-sectional	United States, Canada, and Europe (university veterinary hospitals)	A total of 295 respondents: Case group: 209 Female veterinary anesthesia staff (faculty, residents, interns, technicians) Control Group: 86 female veterinary critical care staff	Discussed in context of NIOSH recommendations	Questionnaire-based survey	Nitrous oxide, isoflurane, and halogenated agents	The study found no statistical difference in the rates of miscarriage, time to conceive, or birth defects between veterinary anesthesia staff (exposed) and veterinary critical care staff (control).	Fertility treatment in some cases
Lawson C. et al. (2012)	Retrospective Cohort	United States	Total of 7,482 Female nurses Control Group: Unexposed nurses within the Nurses’ Health Study II cohort	Self-reported duration (>1 h/day vs. < 1 h/day)	Retrospective questionnaire (Self-reported)	Nitrous oxide, Halothane, Enflurane, Isoflurane	In the analysis of Nurses’ Health Study II, exposure to anesthetic gases was not associated with an increased risk of spontaneous abortion. (Significant risks were found for antineoplastic drugs and sterilizing agents).	Several confounder including smoking, alcohol, caffeine, race and ethnicity
Adeleh Shirangi et al. (2008)	Retrospective Cohort	Australia	Total of 1,197 female veterinarians (analyzing 633 pregnancies) Control Group: Unexposed veterinarians (internal cohort comparison)	Defined by hours per week (>1 h) and presence/absence of scavenging	Mailed questionnaire	Isoflurane, halothane, sevoflurane, N₂O	Found a more than two-fold increase in the risk of spontaneous abortion for female veterinarians exposed to unscavenged anesthetic gases for ≥1 h/week. Whereas, no significant risk was found when scavenging equipment was used.	12 known confounders (not mentioned in details)
Axelsson et al. (1996)	Nested Case–Control	Sweden	Total of 3,985 practicing midwives. Control Group: Midwives with low or no exposure (<10% of deliveries)	Classified by frequency of use (>50% of deliveries vs. < 10%)	Postal questionnaire	Nitrous oxide	Use of Nitrous Oxide (>50% of deliveries) was not associated with an increased risk of spontaneous abortion among Swedish midwives. (Night shifts were identified as the primary risk factor).	Smoking, and work related stress
Rowland et al. (1995)	Retrospective Cohort	United States	Total of 1,465 dental assistants (from a larger survey of 4,856). Expose group: 761 -Control Group: 684 Unexposed dental assistants (or those working in scavenged offices)	>3 h per week (High) vs. None/Low.	Mailed questionnaire	Nitrous oxide	Significant elevation in risk of spontaneous abortion (Relative Risk = 2.6) was found only among dental assistants working with Nitrous Oxide for ≥3 h/week in offices without scavenging equipment.	Smoking, differences in maternal ages, heavy metal exposure (Mercury)
Saurel-cubizolles et al. (1994)	Cross-sectional	France	Total of 417 operating room nurses (describing 776 pregnancies). Control Group: Female nurses in other departments	Work in operating room	Interview	Anesthetic gases (general)	The rate of spontaneous abortion was significantly higher for pregnancies during which nurses worked in an operating room compared to other nursing roles.	Smoking, differences in maternal ages, exposure to X-ray
Guirguis SS et al. (1990)	Retrospective Survey	Canada	Total of 8,538 nurses. Expose group: 6336 Control Group: 2202 Unexposed hospital personnel	Work in operating room or recovery room	Retrospective mail survey	Anesthetic gases	This study generally did not find a strong, statistically significant association between anesthetic gas exposure and spontaneous abortion.	Smoking, differences in maternal ages.
Hemminki Kari et al. (1985)	Retrospective Cohort	Finland	Total of 217 case nurses (with spontaneous abortion). Control Group: Nurses who had a normal birth	Presence of exposure in the first trimester	Register-based study (Hospital Discharge Register) combined with exposure confirmation from head nurses	Anesthetic gases	No significant increase in the risk of spontaneous abortion or malformations was observed among nurses exposed to anesthetic gases.	NR
Heidam, Lene Zeuthen et al. (1984)	Retrospective Cohort	Denmark	Totla cases: 2159 Exposed: 728 Unexposed: 1421 people	Occupational category	Postal questionnaire	Nitrous oxide	No significantly increased risk of spontaneous abortion was linked to Nitrous Oxide exposure compared to controls.	Exposure to heavy metals (mercury)
Axelsson GÖSTAet al (1982)	Cross-sectional	Sweden	Total of 636 nurses Control Group: Unexposed women (non-hospital or non-exposed hospital staff)	High vs. Low/None	Postal questionnaire	Anesthetic gases	Reported a higher miscarriage rate among exposed hospital workers, but the difference was not statistically significant.	NA
Lauwerys Robert et al. (1981)	Retrospective Survey	Belgium	Total of 435 nurses Exposed: 204 Control Group: 231 Unexposed personnel.	Internal dose concentration	Biological monitoring: Measurement of anesthetic agents in urine and exhaled breath.	Anesthetic gases (Nitrous oxide, Halothane)	there was no significant increase in reproductive hazards for medical personnel exposed to anesthetic gases compared to unexposed controls.	Smoking
Cohen Ellis et al. (1980)	Retrospective Survey	United States	Total of 21,202 dental assistants. Control Group: Unexposed dental assistants	“Heavy” exposure defined as >8 h per week	Questionnaire	Chronic exposure to trace anesthetic gases, especially Nitrous oxide	Dental assistants heavily exposed to anesthetic gases (specifically Nitrous Oxide) showed a spontaneous abortion rate 1.7 to 2.3 times higher than unexposed controls.	Smoking, Heavy metal exposure (Mercury)
Cohen Ellis et al. (1971)	Retrospective Survey	United States	Total of 251 operating room personnel, including nurses and anesthesiologists. Control Group: General Duty Nurses	Work in operating room	Questionnaire	General inhalational anesthetics (specific agents not identified)	A significantly higher rate of spontaneous abortion in operating room nurses (29.7%) and anesthetists (37.8%) compared to unexposed controls (~10%).	Smoking and alcohol consumption

### 3.2 Main outcomes

**Figure 2:**
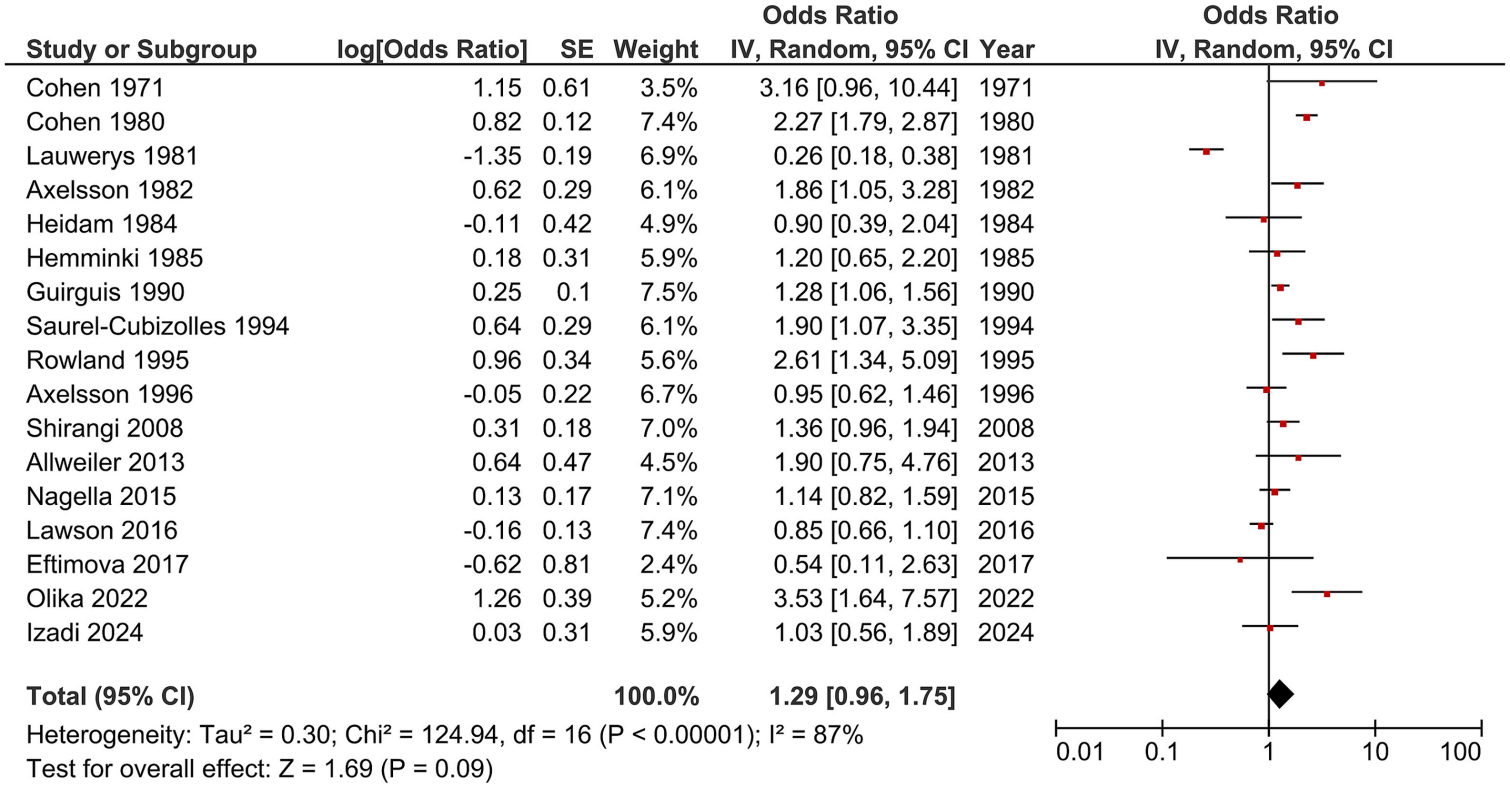
Forest plot of the 17 studies assessing the association between occupational exposure to inhalational anesthetics and spontaneous abortion. The red squares represent the odds ratio (OR) for each study, with horizontal lines indicating the 95% confidence intervals (CI). The black diamond at the bottom represents the overall pooled odds ratio calculated using a random-effects model. There is significant heterogeneity among the included studies.

The included studies reported varying effect sizes and degrees of precision, as shown in the forest plot (Figure 2). Most studies demonstrated an increased risk of spontaneous abortion among healthcare workers exposed to inhalational anesthetics, with several showing statistically significant associations—such as Olika et al. (OR = 3.53, 95% CI 1.64–7.57), Rowland et al. (OR = 2.61, 95% CI 1.34–5.09), Cohen et al. (OR = 2.27, 95% CI 1.79–2.87), Saurel-Cubizolles et al. (OR = 1.90, 95% CI 1.07–3.35), Axelsson et al. (OR = 1.86, 95% CI 1.05–3.28) and Guirguis et al. (OR = 1.28, 95% CI 1.06–1.56).

Other studies—including those by Izadi et al. (2024), Allweiler (2013), Eftimova et al. (2017), Axelsson et al. (1996), Cohen et al. (1971), Lawson et al. (2016), Heidam et al. (1984), Hemminki et al. (1985), Lauwerys et al. (1981), Nagella et al. (2015), and Shirangi et al. (2008)—did not observe statistically significant effects.

The pooled random-effects estimate demonstrated 1.29-fold higher odds of spontaneous abortion among exposed workers compared with non-exposed controls (OR = 1.29, 95% CI 0.96–1.75; Figure 2). These results align with earlier meta-analyses, such as that by Boivin (1), which reported a relative risk of 1.48 for occupational exposure to inhalational anesthetics. However, the results from this study showed the overall estimate is insignificant.

### 3.3 Heterogeneity and biological plausibility

A high degree of heterogeneity was detected across studies (I^2^ = 87%, *p* < 0.00001), indicating substantial variability in true effect sizes. This heterogeneity likely reflects methodological, recall bias in retrospective studies and contextual differences among studies, including variations in study design, participant populations, exposure measurement methods, exposure duration, and the effectiveness of scavenging and ventilation systems. The time period in which the study was conducted seemed to be a significant source of heterogeneity. Studies conducted prior to 1990 likely involved higher ambient anesthetic concentrations due to less effective ventilation technologies. The clinical setting and the type of anesthetic agents (e.g., nitrous oxide, halothane, isoflurane, sevoflurane) can also affect the type and intensity of exposure. Geographic and infrastructural variability (in terms of modern and developing regions) could alter the results.

From a biological standpoint, the findings are supported by mechanistic evidence linking waste anesthetic gas (WAG) exposure to DNA damage and oxidative stress in healthcare workers (2, 30).

Experimental and occupational studies have shown that metabolic by-products of anesthetic agents can generate reactive oxygen species (ROS), leading to oxidative injury to nucleic acids, proteins, and lipids (31). Such cellular and genetic damage may impair gamete integrity or early embryonic development, providing a plausible mechanistic explanation for the observed association with spontaneous abortion (11). It should be mentioned that spontaneous abortion in these workers is likely not caused by a single “toxic dose” of a gas, but the cumulative and even synergistic effect of different chemical and physiological stressors (the exposome model). Epigenetics may play a more subtle but critical role by altering genes that are essential for fetal development (32).

### 3.4 Subgroup analysis

**Figure 3:**
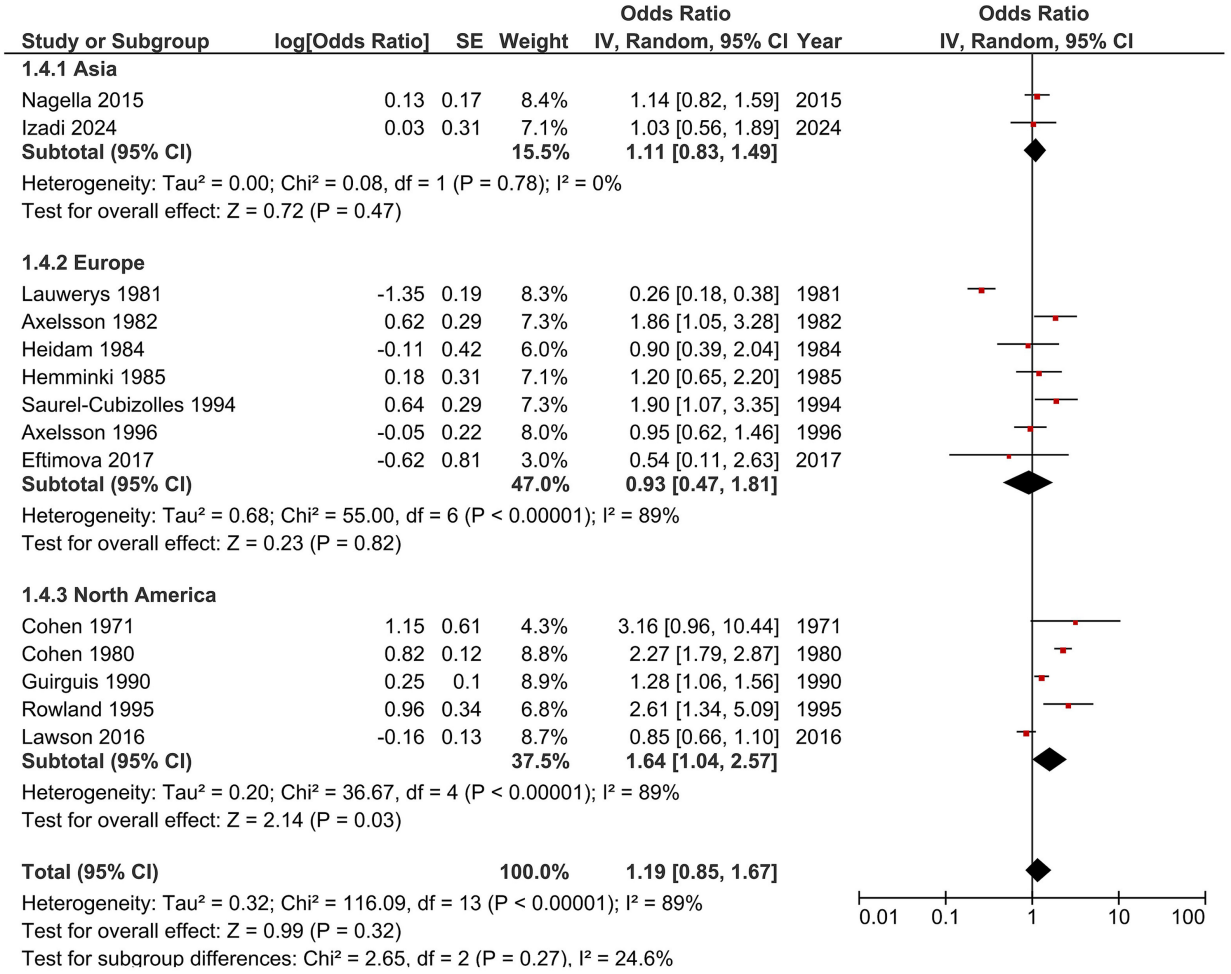
Forest plot of subgroup analysis by geographic region. Pooled odds ratios (ORs) for studies from Asia, Europe, and North America are shown. Studies from North America showed a stronger and significant relationship (OR = 1.64, 95% CI 1.04–2.57). However, the regional factors did not statistically influence the overall effect among subgroup (*p* = 0.27; *I*^2^ = 24.6%).

**Figure 4:**
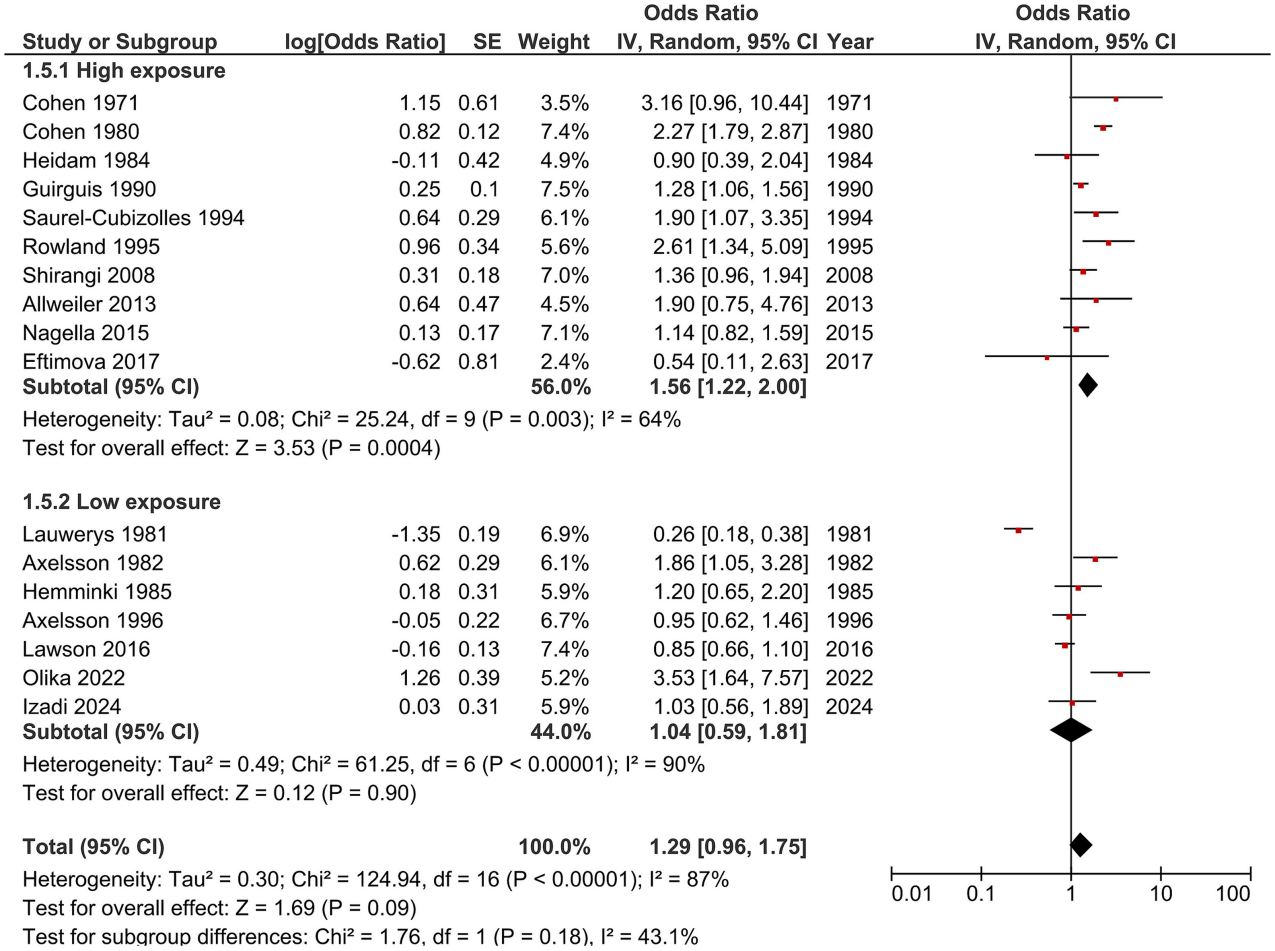
Forest plot of subgroup analysis by exposure level. Pooled odds ratios (ORs) for high-exposure and low-exposure healthcare workers, showing the association between anesthetic gas exposure and spontaneous abortion. The combined analysis of all 17 studies indicates a non-significant trend toward increased risk. The subgroup test suggests that the low-exposure group shows an insignificant effect, whereas the high-exposure group shows a stronger and significant effect. The difference between the two subgroups is not statistically significant.

Subgroup analyses were conducted to explore potential sources of heterogeneity (Figures 3, 4). When stratified by geographic region, pooled estimates varied modestly (Figure 3). Studies from Asia (OR = 1.11, 95% CI 0.83–1.49) and Europe (OR = 0.93, 95% CI 0.47–1.81) showed associations close to the null, whereas studies from North America indicated a stronger relationship (OR = 1.64, 95% CI 1.04–2.57). However, subgroup differences were not statistically significant (*p* = 0.27; I^2^ = 24.6%), suggesting that regional factors did not substantially influence the overall effect.

When stratified by exposure level, studies involving high-exposure healthcare workers—such as operating room nurses, anesthesiologists, dental staff, and veterinarians—showed a significantly increased risk (OR = 1.56, 95% CI 1.22–2.00; Figure 4). In contrast, studies involving low- or mixed-exposure workers, including ward nurses, recovery room staff, and midwives, showed weaker, non-significant associations (pooled OR = 1.04, 95% CI 0.59–1.81). The subgroup difference was not statistically significant (*p* = 0.18; I^2^ = 43.1%). These findings suggest that while high exposure may contribute to increased risk, exposure intensity alone does not fully explain the observed heterogeneity.

Overall, the subgroup results indicate that variability across studies is unlikely to be driven solely by regional or occupational exposure differences. Instead, factors such as exposure duration, ventilation quality, and protective practices likely play combined roles.

### 3.5 Meta-regression analysis

Random-effects meta-regressions were performed to explore potential sources of heterogeneity among the included studies. When region was entered as a categorical moderator, no statistically significant association was found between geographic location and effect size (*p* = 0.46), and the model explained little of the between-study heterogeneity (R^2^ = 0%). Similarly, subgroup analyses by profession (anesthesiologists, dental staff, and nurses), exposure level (high vs. low/mixed), and type of anesthetic agent (nitrous oxide or mixed gases) did not reveal significant subgroup differences, indicating that these variables alone did not account for the observed heterogeneity. In contrast, exposure duration emerged as the most influential variable, showing a significant positive correlation with the effect size. When exposure duration (hours) was analyzed as a single moderator, a significant positive relationship was observed. (coefficient = 0.105, SE = 0.041, *p* = 0.010), indicating that each additional hour of exposure increased the odds of spontaneous abortion by approximately 11%. This model explained 73.6% of between-study heterogeneity (R^2^ = 73.6%) and reduced the residual I^2^ to 62.6%.

When both exposure duration and exposure level were included simultaneously, neither remained significant (duration coefficient = 0.081, *p* = 0.197; exposure coefficient = −0.276, *p* = 0.528). The explained heterogeneity declined to 52.7% (R^2^ = 52.7%), and residual I^2^ remained moderate (73.2%). Although based on a limited number of studies (*n* = 4), these findings highlight exposure duration as the main determinant of heterogeneity, whereas exposure intensity and other study-level factors appeared less influential.

### 3.6 Sensitivity analysis

Leave-one-out sensitivity analysis confirmed the robustness of the pooled estimates. The overall pooled odds ratio was 1.29 (95% CI 0.95–1.74, *p* = 0.103). Sequential omission of individual studies yielded consistent results, with pooled ORs ranging from 1.22 (when Olika 2022 was omitted) to 1.42 (when Lauwerys 1981 was omitted). All models produced overlapping confidence intervals, indicating that no single study had an undue influence on the overall findings.

### 3.7 Publication and risk of bias and quality assessment

**Figure 5:**
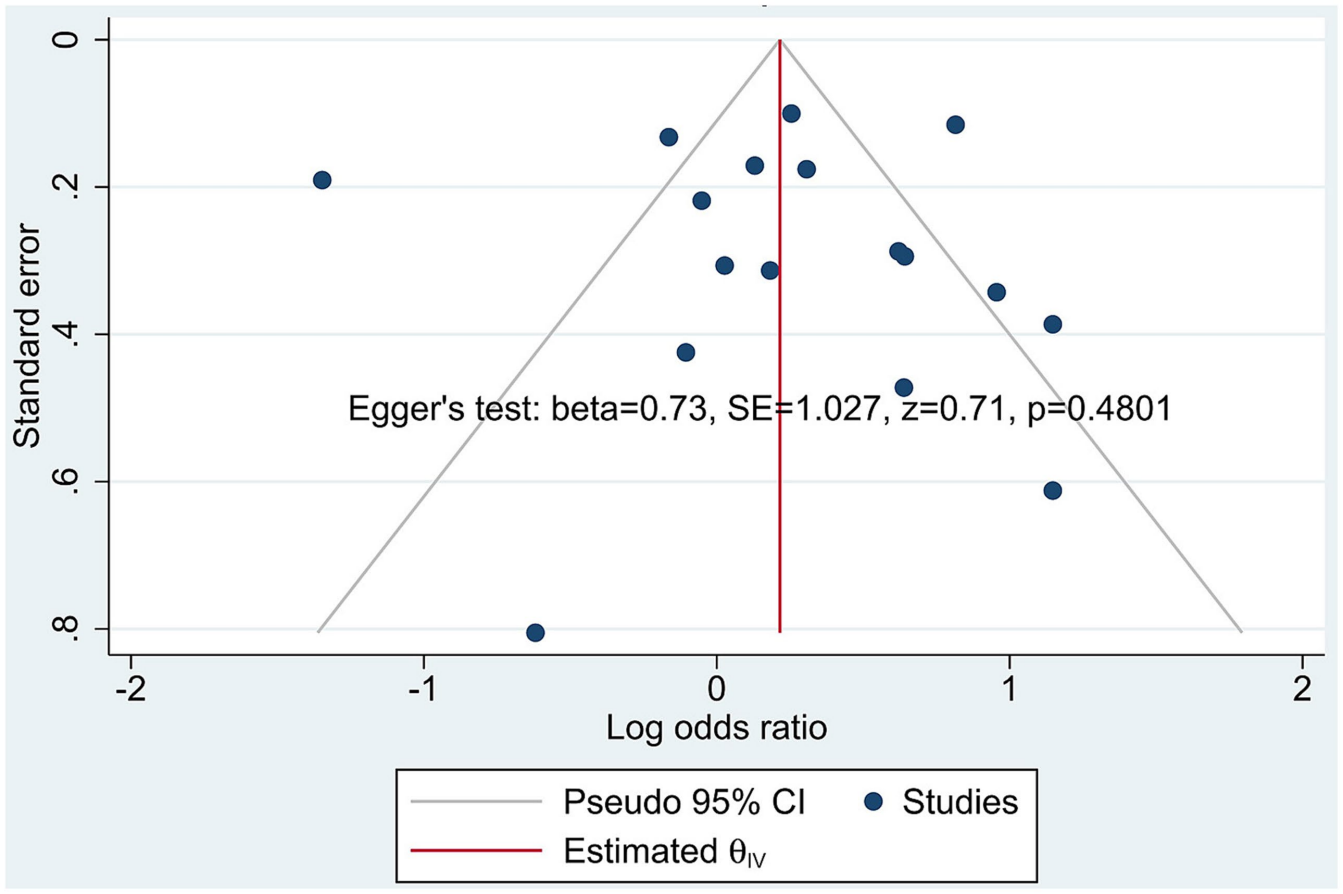
Funnel plot assessing potential publication bias and small-study effects across studies included in the meta-analysis. The funnel plot illustrates the distribution of individual studies (blue dots) by plotting the standard error against the log odds ratio. The embedded Egger’s test results (beta = 0.73, SE = 1.027, *z* = 0.71, *p* = 0.4801) indicate no statistically significant evidence of publication bias in this meta-analysis.

**Figure 6:**
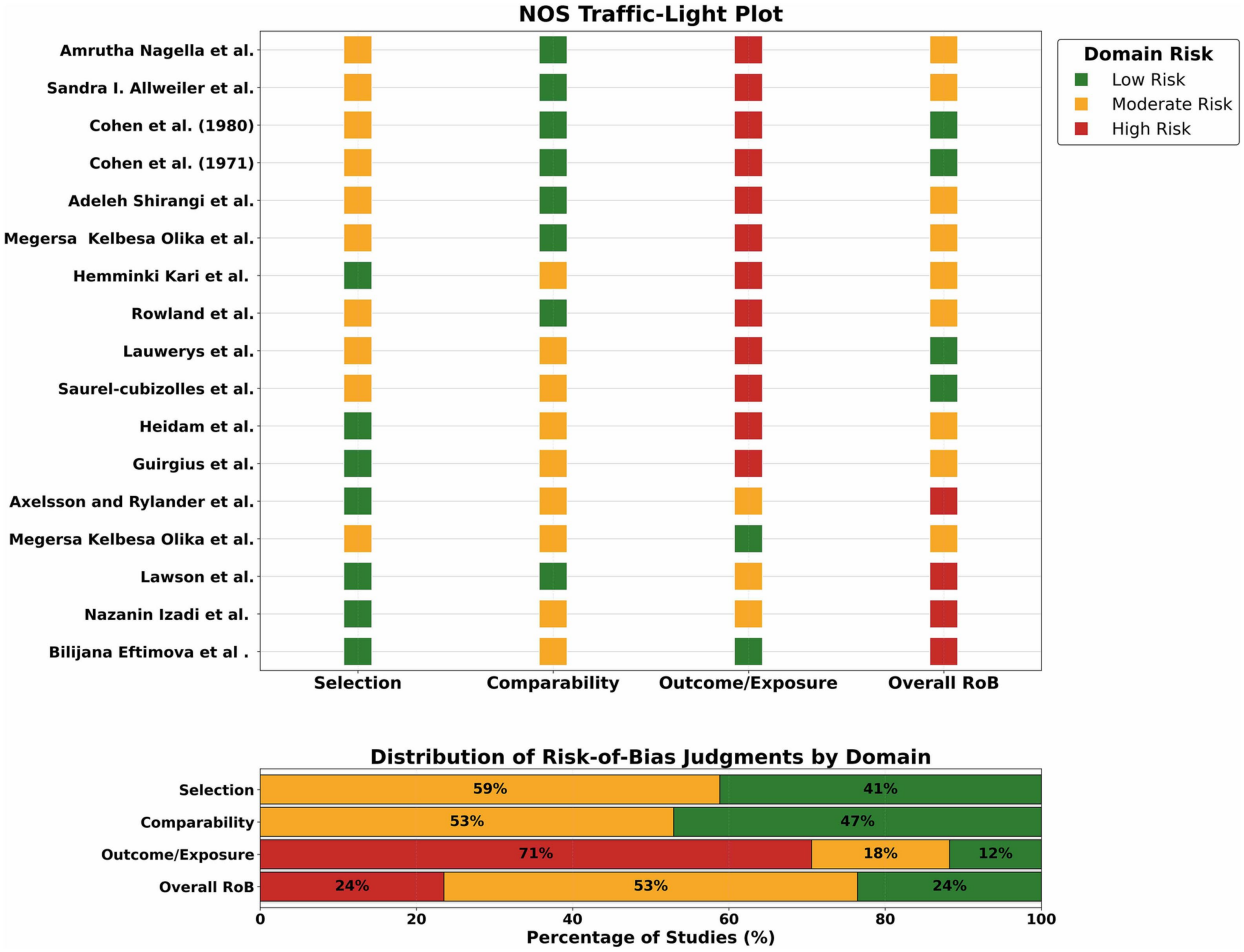
Traffic light plot showing results of quality assessment using the NOS instrument.

Publication bias was evaluated both visually and statistically. The funnel plot (Figure 5) displayed a generally symmetrical distribution of studies around the pooled estimate, suggesting minimal small-study effects. Egger’s regression test (*β* = 0.73, SE = 1.03, z = 0.71, *p* = 0.48) did not indicate statistically significant publication bias, supporting the conclusion that selective reporting is unlikely to have meaningfully influenced the results. The results for risk of bias assessment, using the NOS instrument, are shown in Figure 6.

## 4 Discussion

The systematic review and meta-analysis were conducted to assess the association between inhalation anesthetic and the risk of spontaneous abortion in women occupationally exposed to these gases. This study finds a significant positive association between risk of spontaneous abortion and occupational exposure to inhalation anesthetics. Our primary finding, a pooled odds ratio of 1.29 (95% CI 0.96–1.75), aligns with historical data such as Boivin’s 1997 analysis (1), even though this increase was not significant in our study, despite study by Boivin. It also confirms that, irrespective of contemporary practices, the risk prevails and is actually greater in some studies than acknowledged (33). It should be noted that the risk of spontaneous abortion was significantly increased in health workers that had been highly exposed to anaesthetic gases (OR = 1.56, 95% CI 1.22–2.00).

Research regarding the potential impact of exposure to inhalation anesthetics in the workplace on reproductive outcomes has been conducted for decades (34). Early epidemiological studies, many of which were completed prior to the implementation of scavenging systems for operating rooms, suggested a link between potential reproductive risk and occupational exposure to inadvertent anesthetic gases in women with evidence of an increased risk for spontaneous abortion (35, 36). Scavenging systems are designed to minimize healthcare workers’ occupational exposure to inhalation of anesthetics because they remove anesthetic gases from the air in the operating room. Occupational exposure and other related health risks, including the risk of spontaneous abortion associated with exposure to the occupational inhalation anesthetics, are thus reduced (37). These results are important because they add biological plausibility, as DNA damage caused by waste anesthetic gases is known to be a precursor to serious long-term reproductive and developmental consequences such as congenital defects, premature births, miscarriages, and infertility (7). Due to scavenging and ventilation now being permissible and significant decreases in ambient levels of anesthetic gas, some more recent studies have cautiously suggested there is no longer a significant difference between contemporary occupational exposure and rates of spontaneous abortion (7, 34). Specifically, a 2023 systematic review that concluded modern scavenged systems had effectively eliminated reproductive risks (8). However, our analysis suggests that despite widespread implementation of scavenging technologies, a significant risk persists. Notably, the cumulative role of different variables other than a single agent (the exposome model) and epigenetic changes needs to be considered (32).

The meta-regression and subgroup analyses shed light on potential sources of heterogeneity across studies. Geographic region, professional group, exposure level, and anesthetic agent were not significantly associated with the pooled effect estimates, indicating that variations in study outcomes are unlikely to be explained by these contextual factors alone. In contrast, exposure duration emerged as the most influential moderator, showing a significant positive association with the effect size. This is a factor that is often overlooked in binary “exposed vs. non-exposed” comparisons. Each additional hour of exposure corresponded to an estimated 11% increase in the odds of spontaneous abortion, and this variable explained more than 70% of the between-study heterogeneity. Although the association attenuated when exposure level was included in the model, likely due to overlapping exposure categories and the limited number of studies, exposure time consistently accounted for a substantial portion of variability. These findings suggest that prolonged or cumulative exposure to anesthetic gases may have a dose-dependent effect on reproductive risk, consistent with prior evidence linking longer occupational exposure to adverse reproductive and neurological outcomes. Overall, the results demonstrate that the risk is exposure level-dependent and therefore, it challenges the safety of current exposure controls in entirely eliminating reproductive hazards.

The level of risk, however, available in WAGs, is affected greatly by exposure characteristics such as concentration and duration of exposure to WAGs. Many studies reported in previous literature have reported WAGs at levels in excess of the recommended occupational exposure limits of the NIOSH (38, 39). However, The American Conference of Governmental Industrial Hygienists (ACGIH) suggests a higher guideline of 50 ppm for both nitrous oxide and halogenated agents (33). Internationally, limits for nitrous oxide are generally higher; the United Kingdom and several European nations (including Germany and Sweden) allow up to 100 ppm, while Australia follows a stricter 25 ppm limit similar to NIOSH. For halogenated agents, international limits typically range between 10 and 50 ppm depending on the specific gas, though newer agents like Sevoflurane and Desflurane often do not have a specific regulatory limit (33)The increasing occurrence of exposure at rates above recommended exposure limits creates an ongoing occupational risk, particularly in healthcare facilities without equipment for proper scavenging and ventilation, or those that do not maintain the equipment. Further research has indicated that chronic exposure over an average of 3 to 19 years represents links for multiple factors of DNA damage (40). There is also evidence that this previously limited exposure has a cumulative effect, increasing the risk of possible adverse health outcomes. A key finding of this study is the statistical significance of exposure level (high vs. low) over exposure intensity in explaining the heterogeneity. However, this relationship requires careful interpretation. While duration indicates the cumulative load, the level of exposure still remains the biological factor for the mechanism of damage. High-concentrations, even in short period, can interrupt metabolic pathways and the DNA repair mechanisms, leading to the genotoxicity and oxidative stress (ROS) described in the literature (41–43). Furthermore, the complexity of exposure cannot be measured solely through ambient air. As highlighted by recent toxicological studies, the interval between consecutive exposure periods is crucial (44). If the interval between shifts is insufficient, the body does not adequate recovery time to metabolize bioaccumulation may occur. A previous study demonstrated even in modern hospitals equipped with high levels of scavenging and ventilation systems, metabolites of sevoflurane are still detectable in the urine of healthcare workers the day after exposure, suggesting a potential accumulation of toxic substances (45). The current results suggest that attention may be required to meet recommended exposure limits and maintain operating room air quality to detect any malicious leaks or faults (39).

To address the identified risk of spontaneous abortion, as well as other possible consequences, the aforementioned preventative actions are essential. The first and foremost action must be the installation and rigorous establishment of effective scavenging systems for operating rooms, which are essential for reducing ambient levels of WAGs (34, 35). Other actions are using low fresh flow, using more intravenous anesthetics, and adequately minimizing, if possible, eliminating nitrous oxide. Developing routine surveillance programs to monitor operating room air quality and measure worker exposure to WAGs is important to identify hazards at an early point. Biological monitoring performed, both before and at the end of the work shift, can assess the exact dose and verify whether the anesthetic agents have been adequately cleared from the body. This approach provides a more accurate image of individual physiological burden than ambient ppm levels alone. In addition, the use of antioxidant supplementation for operating room workers may be a means of protecting against damage, given that oxidative stress is part of the process associated with WAG-induced DNA damage (46). Addressing the obstacles to the delivery of these controls, particularly in low-resource environments where exposure controls may be inadequate, is an important public health issue (39, 41).

Considering the persistent risk of anesthetic gas even in modern settings, the passive safety systems seem inadequate. Our results underscore the importance of active, daily vigilance. Installing scavenging systems are not simply enough; their functional integrity must be also verified. We emphasize on the need for routine checks of anesthesiologic procedures and equipment. This includes regular inspections for “micro-leaks” that contribute to chronic low-level exposure leaks. Furthermore, healthcare workers must go through continuous staff training not only on the operation of control equipment but also on the long-term health effects of exposure and adhere to best practices (e.g., proper mask fit, avoiding high flows).

### 4.1 Limitations

This study was influenced by certain limitations. First, while we identified exposure duration as a key moderator, many included studies relied on retrospective self-reporting, a potential for recall bias. Second, high heterogeneity remained in some analyses, representing potential confounders such as specific genetic polymorphisms in detoxification enzymes or co-exposure to other occupational stressors (the exposome) that may have affected the outcomes. It is of note to say that. The definition of abortion (e.g., <12 weeks versus <20 weeks) could be another source of heterogeneity. Third, most primary studies did not differentiate between specific gases (e.g., nitrous oxide vs. halogenated agents), preventing a more accurate analysis. Another limitation is that it would be better if we used more relevant risk of bias criteria, however the poor knowledge of authors about this criterion was an obstacle. Finally, the lack of standardized biological monitoring data across the included studies limits our ability to correlate the observed risk directly with exact biological doses. Future studies, particularly dose–response meta-analysis are necessary to address these gaps.

Future studies should focus on prospective cohort studies with a higher sample size in order to establish more robust causal relationships and further clarify dose–response metrics. Future studies should also include research on the specific molecular pathways involved in WAG-induced DNA damage and oxidative stress, which allow for better characterization of WAGs as a health hazard in order to derive specific interventions. Further research should also address planned evaluations of the efficacy of various exposure control strategies in different healthcare settings and across varied socioeconomic environments. The potential combined effects of exposure to WAGs with other occupational hazards (for example, ionizing radiation from surgical approaches) need to be explored.

## 5 Conclusion

In summary, our meta-analysis supports that occupational exposure to inhalation anesthetic agents could increase the risk of spontaneous abortion, one of the most serious adverse effects on reproductive health in healthcare workers. While this increase was significant in individuals highly exposed to inhalation anesthetic agents, it was not significant in those with low exposure. Therefore, the exposure level, including intensity, duration, and interval, are fundamental factors in controlling the adverse effects among workers, as demonstrated by the dose–response relationship. This result, coupled with our evidence of WAG-induced DNA damage, argues for a reiterated effort and commitment to stringent exposure control, strong monitoring programs, and sufficient occupational health policies. Protection of these essential workers mandates collaboration across stakeholders to represent and advocate that these workers have safe working environments to promote an injury-free reproductive status.

## Data Availability

The original contributions presented in the study are included in the article/Supplementary material, further inquiries can be directed to the corresponding author.
