# Supplementary material for: Occupational exposure to inhalational anesthetics and the risk of spontaneous abortion: a systematic review and meta-analysis
Source: Front Public Health. 2026 Apr 1;14:1766912. doi: 10.3389/fpubh.2026.1766912 (PMC13080247; doi:10.3389/fpubh.2026.1766912)
Supplement: Supplementary file 1 [file Supplementary_file_1.docx]

**Detailed Search Keyword**

**Web of Sciences : 122 results**

TS=("anesthetic gases" OR "anaesthetic gases" OR "inhalational anesthetics" OR "inhalational anaesthetics" OR "waste anesthetic gases" OR "nitrous oxide" OR halothane OR isoflurane OR sevoflurane OR desflurane OR enflurane) AND TS=("spontaneous abortion" OR miscarriage OR "reproductive outcomes" OR "congenital abnormalities" OR teratogenesis)

**PubMed : 145 studies**

("anesthetic gases"[tiab] OR "anaesthetic gases"[tiab] OR "inhalational anesthetics"[tiab] OR "inhalational anaesthetics"[tiab] OR "waste anesthetic gases"[tiab] OR "Nitrous Oxide"[Mesh] OR "Anesthetics, Inhalation"[Mesh] OR nitrous oxide[tiab] OR halothane[tiab] OR isoflurane[tiab] OR sevoflurane[tiab] OR desflurane[tiab] OR enflurane[tiab]) AND ("Abortion, Spontaneous"[Mesh] OR "spontaneous abortion"[tiab] OR miscarriage[tiab] OR "reproductive outcomes"[tiab] OR "congenital abnormalities"[tiab] OR teratogenesis[tiab])

**Scopus: 451**

( TITLE-ABS-KEY ( "anesthetic gases" OR "anaesthetic gases" OR "inhalational anesthetics" OR "inhalational anaesthetics" OR "waste anesthetic gases" OR "nitrous oxide" OR halothane OR isoflurane OR sevoflurane OR desflurane OR enflurane ) ) AND ( TITLE-ABS-KEY ( "spontaneous abortion" OR miscarriage OR "reproductive outcomes" OR "congenital abnormalities" OR teratogenesis ) )
